# Supplementary material for: A non-spatial account of place and grid cells based on clustering models of concept learning
Source: Nat Commun. 2019 Dec 12;10:5685. doi: 10.1038/s41467-019-13760-8 (PMC6908717; doi:10.1038/s41467-019-13760-8)
Supplement: Supplementary file 1 — Supplementary Information [file 41467_2019_13760_MOESM1_ESM.pdf]

Supplementary Information

A non-spatial account of place and grid cells based on clustering models of concept learning

Robert M. Mok[1*], Bradley C. Love[1,2*]

Affiliations:

[1]Department of Experimental Psychology, University College London, 26 Bedford Way, London, WC1H 0AP, United Kingdom.

[2]The Alan Turing Institute, United Kingdom

*Correspondence to: robert.mok@ucl.ac.uk, b.love@ucl.ac.uk

SUPPLEMENTARY NOTE 1

Comparable grid-like map proportions with empirical data

Our simulations produce activations maps that emulate spatial cells in the mEC with multi-peaks spatial fields, so the appropriate test for the proportion of grid cells is the number of grid cells relative to the total number of spatial cells with multiple peaks. Most studies report percentage of grid cells in relation to all cell types (including head-direction cells, border cells, etc.), but only a few reported and quantified the number of non-grid spatial cells, or if they are multi-peaked or not.

Krupic et al.[1] tested rodents in a square environment and reported the percentage of non-grid spatial cells relative to grid cells, and also the non-grid spatial cells that were periodic using a Fourier analysis. In the mEC population they found 26% grid cells, and 44% non-grid spatial cells with multiple peaks, which means they found (26/(26+44))x100 = 37% grid cells relative to non-grid spatial cells. Using the Fourier analysis method on only spatial and head-direction cells (ignoring other cells in the population), they found 35% grid cells and 43% non-grid periodic spatial cells, and 2% conjunctive grid cells. This amounts to (35/(35+43))x100 = 45%, or (37/(37+43))x100 = 46% grid cells with respect to non-grid periodic grid cells, matching to our 45.3% value in the square environment. Perez-Escobar et al.[2] tested rodents in a circular environment and also report the number of non-grid spatial cells, finding 139 grid cells and 226 non-grid spatial cells, meaning they found (139/(139+226))*100 = 38% grid cells, matching our 38.6% value in the circular environment.

The percentage of grid-like cells show very little difference when the parameters are altered, such as a slower or faster learning rate, or an increase or reduction of the batch size. These results are provided in the code and simulated data.

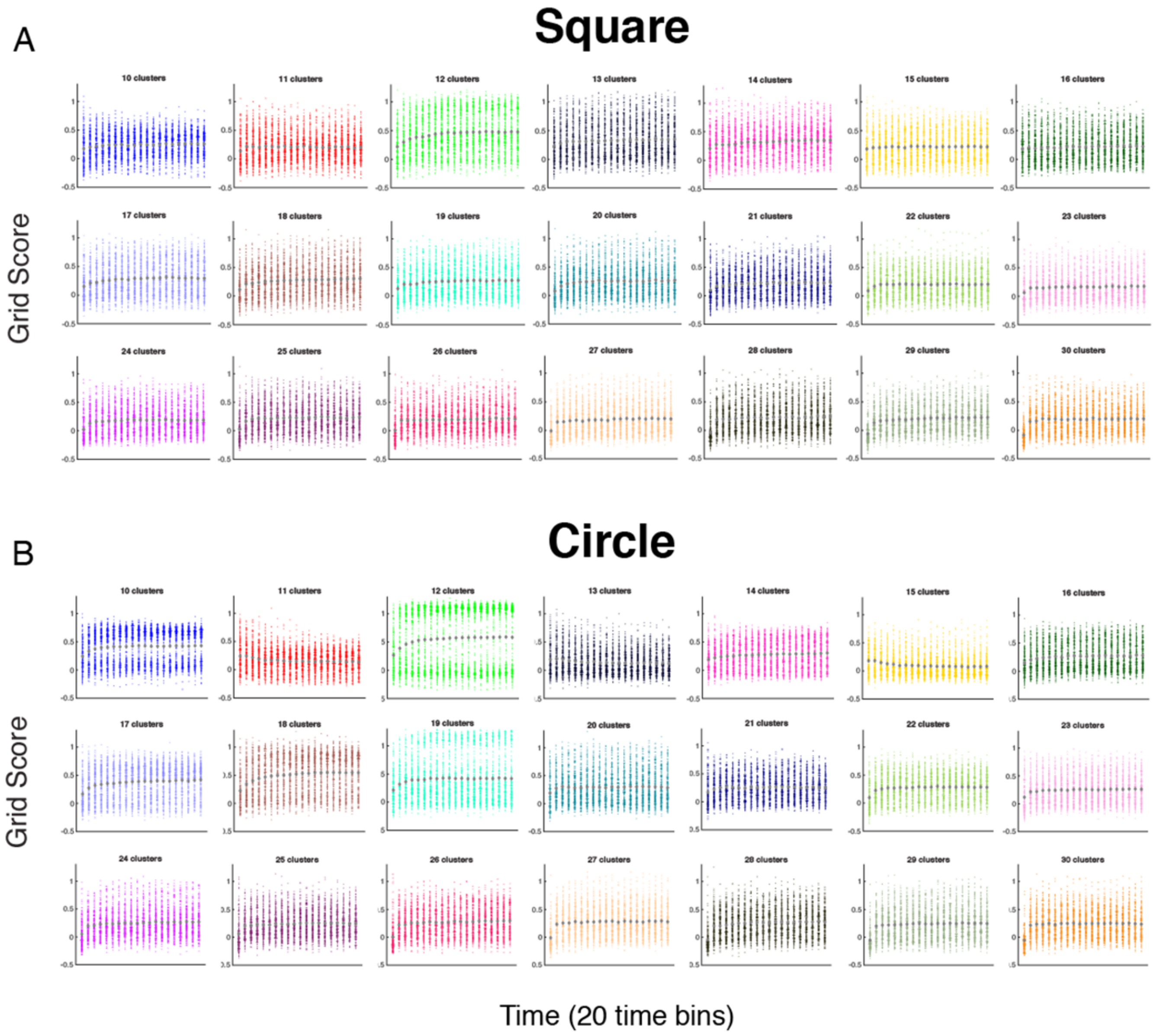

Supplementary Figure 1. Univariate scatterplots showing grid scores increasing over learning in the square (A) and circle (B) for all conditions.

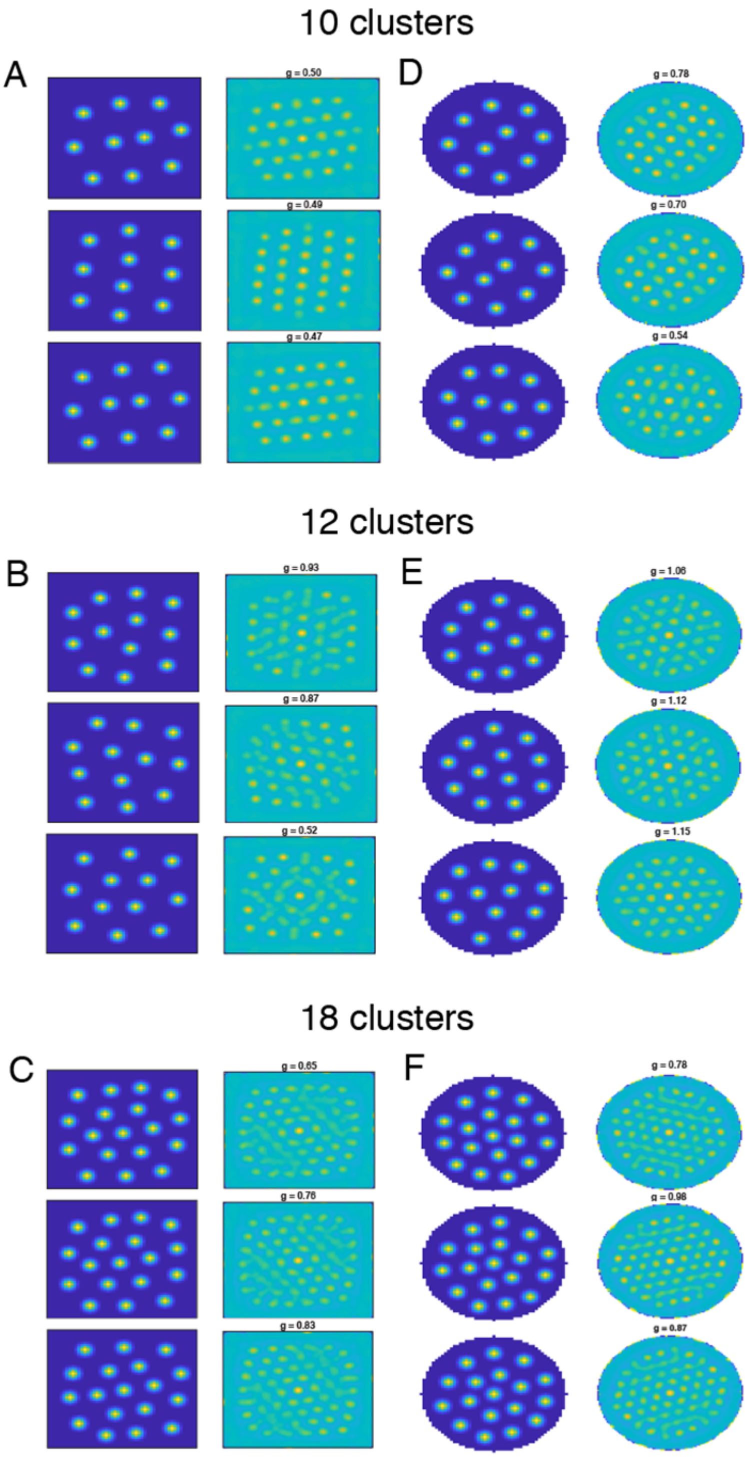

Supplementary Figure 2. Examples of activation maps with grid patterns (left) and their corresponding spatial autocorrelograms (right) in square (A-C) and circular (D-F) environments with 10, 12, and 18 clusters.

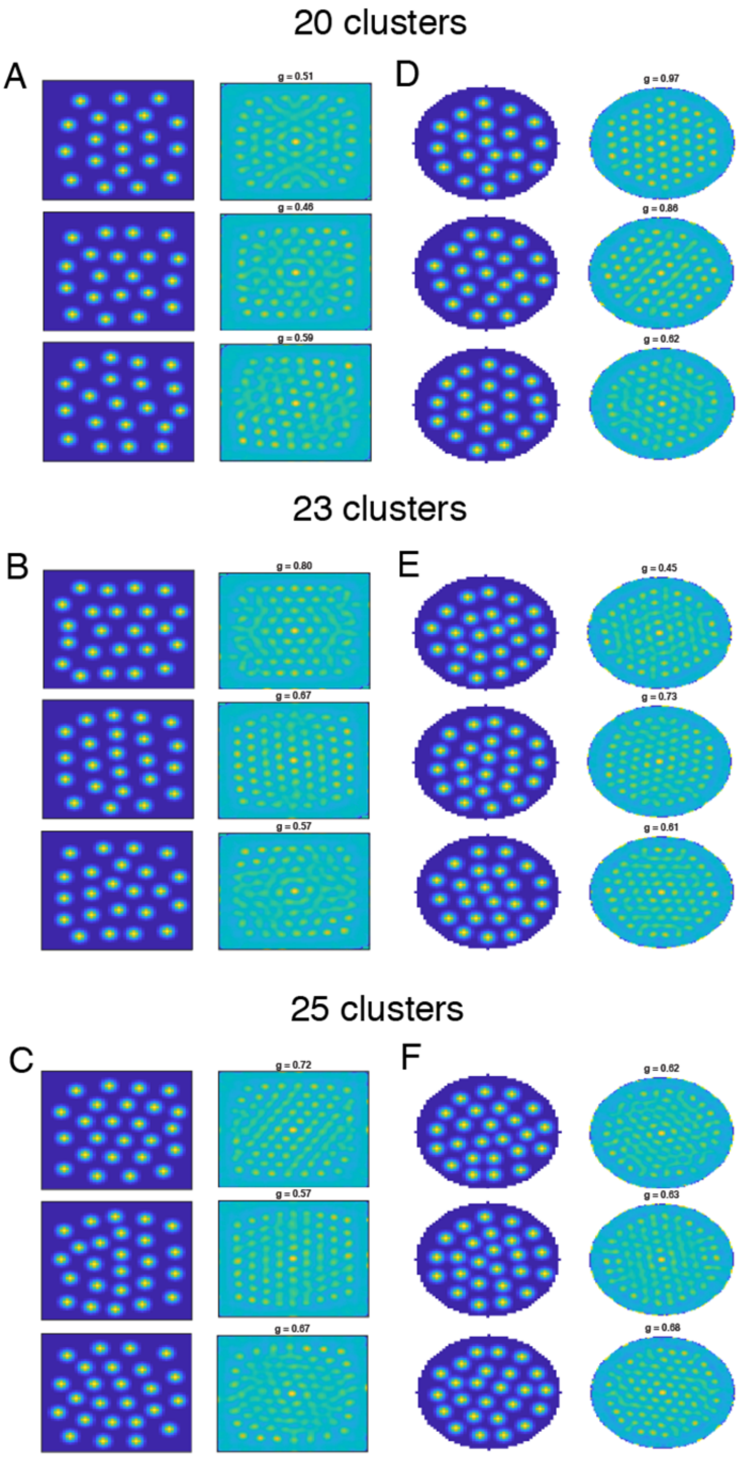

Supplementary Figure 3. Examples of activation maps with grid patterns (left) and their corresponding spatial autocorrelograms (right) in square (A-C) and circular (D-F) environments with 20, 23, and 25 clusters.

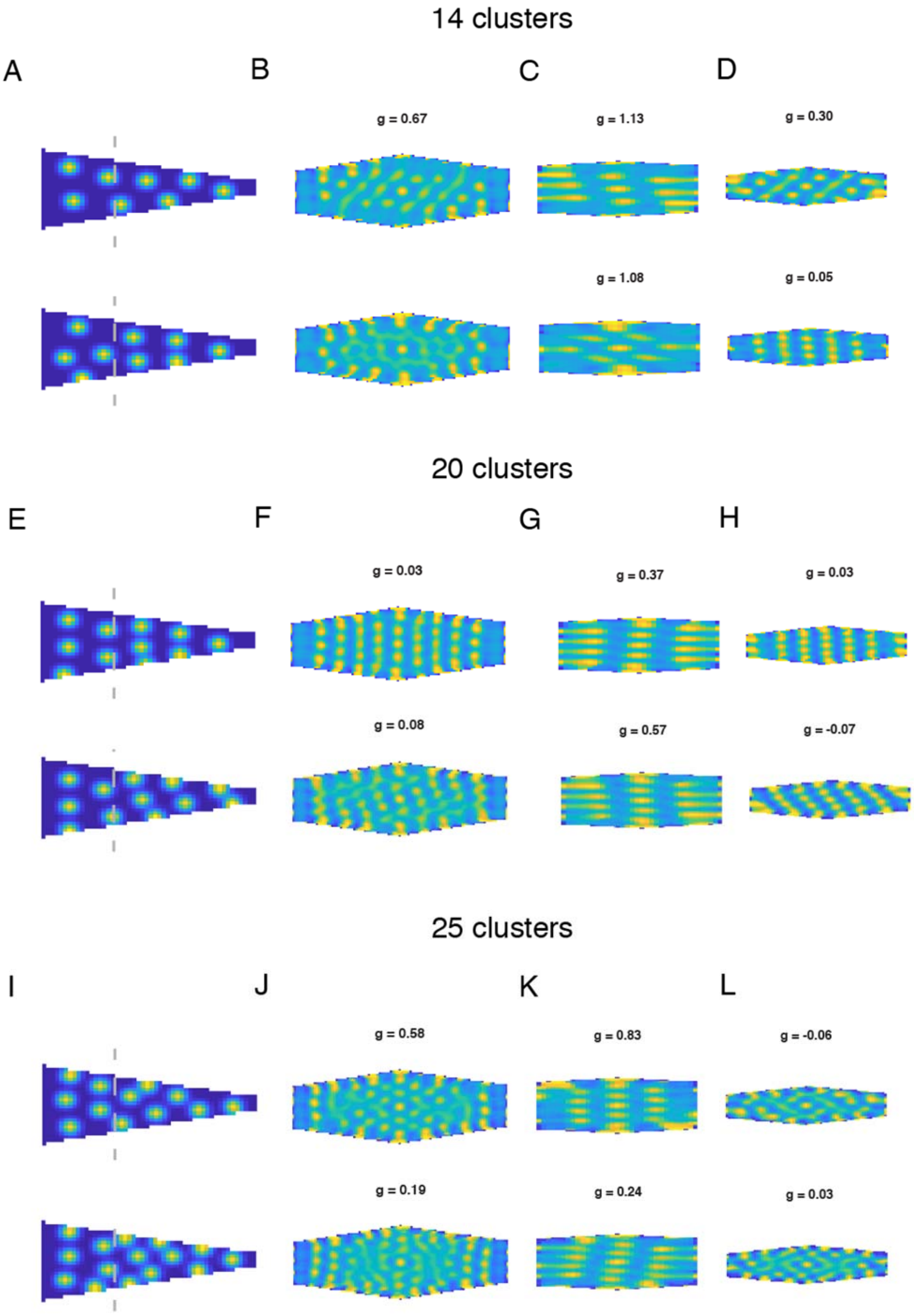

Supplementary Figure 4. Examples of activation maps in the trapezoid environment and their corresponding spatial autocorrelograms with (A-D) 14, (E-H) 20, and (I-L) 25 clusters. (A) Activation map with 14 clusters, (B) spatial autocorrelogram of the full

trapezoid, (C) spatial autocorrelogram of the wide (left) half of the trapezoid, and (D) spatial autocorrelogram of the narrow (right) half of the trapezoid. Same conventions in (E-H) and (I-L).

	Mean	bootstrap CIs
10 clusters	0.0032	[0.0017, 0.0050]
11 clusters	0.0003	[-0.0011, 0.0021]
12 clusters	0.0097	[0.0075, 0.0123]
13 clusters	0.0041	[0.0020, 0.0060]
14 clusters	0.0058	[0.0037, 0.0083]
15 clusters	0.0011	[-0.0006, 0.0031]
16 clusters	0.0021	[-0.0001, 0.0035]
17 clusters	0.0053	[0.0038, 0.0071]
18 clusters	0.0060	[0.0041, 0.0080]
19 clusters	0.0048	[0.0028, 0.0070]
20 clusters	0.0051	[0.0035, 0.0073]
21 clusters	0.0044	[0.0029, 0.0067]
22 clusters	0.0020	[0.0005, 0.0038]
23 clusters	0.0027	[0.0010, 0.0043]
24 clusters	0.0035	[0.0024, 0.0051]
25 clusters	0.0046	[0.0030, 0.0064]
26 clusters	0.0045	[0.0032, 0.0059]
27 clusters	0.0056	[0.0040, 0.0073]
28 clusters	0.0059	[0.0045, 0.0077]
29 clusters	0.0072	[0.0058, 0.0088]
30 clusters	0.0052	[0.0038, 0.0065]

Supplementary Table 1. Mean slopes bootstrap confidence intervals (CIs) for learning over time in the square environment for each condition.

	Mean	bootstrap CIs
10 clusters	0.0051	[0.0035, 0.0066]
11 clusters	-0.0044	[-0.0060, -0.0032]
12 clusters	0.0104	[0.0081, 0.0131]
13 clusters	-0.0038	[-0.0047, -0.0024]
14 clusters	0.0044	[0.0031, 0.0059]
15 clusters	-0.0048	[-0.0060, -0.0033]
16 clusters	0.0042	[0.0029, 0.0059]
17 clusters	0.0081	[0.0065, 0.0096]
18 clusters	0.0113	[0.0091, 0.0135]
19 clusters	0.0056	[0.0033, 0.0084]
20 clusters	0.0019	[0.0003, 0.0039]
21 clusters	0.0017	[0.0003, 0.0033]
22 clusters	0.0042	[0.0026, 0.0057]
23 clusters	0.0038	[0.0023, 0.0052]
24 clusters	0.0057	[0.0040, 0.0073]
25 clusters	0.0043	[0.0031, 0.0057]
26 clusters	0.0067	[0.0053, 0.0081]
27 clusters	0.0057	[0.0045, 0.0069]
28 clusters	0.0075	[0.0059, 0.0091]
29 clusters	0.0060	[0.0046, 0.0073]
30 clusters	0.0050	[0.0032, 0.0061]

Supplementary Table 2. Mean slopes bootstrap CIs for learning over time in the circular environment for each condition.

	Mean	bootstrap CIs
10 clusters	0.2291	[0.2162, 0.2414]
11 clusters	0.1821	[0.1679, 0.1976]
12 clusters	0.4685	[0.4478, 0.4905]
13 clusters	0.3755	[0.3541, 0.3943]
14 clusters	0.3554	[0.3407, 0.3702]
15 clusters	0.2205	[0.2037, 0.2373]
16 clusters	0.2130	[0.1952, 0.2301]
17 clusters	0.2891	[0.2703, 0.3068]
18 clusters	0.3544	[0.3370, 0.3734]
19 clusters	0.2979	[0.2789, 0.3149]
20 clusters	0.2941	[0.2760, 0.3102]
21 clusters	0.2398	[0.2245, 0.2551]
22 clusters	0.2315	[0.2167, 0.2483]
23 clusters	0.2118	[0.1967, 0.2288]
24 clusters	0.2315	[0.2166, 0.2473]
25 clusters	0.2568	[0.2398, 0.2738]
26 clusters	0.2644	[0.2492, 0.2834]
27 clusters	0.2729	[0.2559, 0.2898]
28 clusters	0.2862	[0.2702, 0.3040]
29 clusters	0.2785	[0.2621, 0.2933]
30 clusters	0.2549	[0.2390, 0.2692]

Supplementary Table 3. Mean grid scores and bootstrap CIs in the square environment for each condition.

	Mean	bootstrap CIs
10 clusters	0.4154	[0.3961, 0.4339]
11 clusters	0.1071	[0.0961, 0.1184]
12 clusters	0.5691	[0.5320, 0.6081]
13 clusters	0.1003	[0.0893, 0.1133]
14 clusters	0.2975	[0.2803, 0.3147]
15 clusters	0.0896	[0.0776, 0.1022]
16 clusters	0.2859	[0.2693, 0.3050]
17 clusters	0.4905	[0.4699, 0.5086]
18 clusters	0.5854	[0.5625, 0.6063]
19 clusters	0.4141	[0.3901, 0.4424]
20 clusters	0.3430	[0.3238, 0.3623]
21 clusters	0.2894	[0.2720, 0.3060]
22 clusters	0.2893	[0.2737, 0.3060]
23 clusters	0.2854	[0.2686, 0.3008]
24 clusters	0.2767	[0.2624, 0.2933]
25 clusters	0.3013	[0.2851, 0.3171]
26 clusters	0.3036	[0.2876, 0.3203]
27 clusters	0.3029	[0.2887, 0.3197]
28 clusters	0.2944	[0.2792, 0.3102]
29 clusters	0.2873	[0.2715, 0.3022]
30 clusters	0.2519	[0.2369, 0.2674]

Supplementary Table 4. Mean grid scores and bootstrap CIs in the circular environment for each condition.

	Mean	bootstrap CIs
10 clusters	0.0293	[0.0118, 0.0470]
11 clusters	-0.0097	[-0.0263, 0.0054]
12 clusters	-0.0871	[-0.0997, -0.0745]
13 clusters	-0.1177	[-0.1302, -0.1035]
14 clusters	-0.0369	[-0.0550, -0.0204]
15 clusters	0.1096	[0.0921, 0.1282]
16 clusters	0.1581	[0.1414, 0.1774]
17 clusters	0.1074	[0.0912, 0.1233]
18 clusters	0.0678	[0.0525, 0.0837]
19 clusters	0.0309	[0.0161, 0.0466]
20 clusters	0.0322	[0.0181, 0.0468]
21 clusters	0.0332	[0.0179, 0.0505]
22 clusters	0.0459	[0.0279, 0.0633]
23 clusters	0.0568	[0.0385, 0.0751]
24 clusters	0.0916	[0.0723, 0.1094]
25 clusters	0.0981	[0.0793, 0.1143]
26 clusters	0.1095	[0.0902, 0.1272]
27 clusters	0.1250	[0.1092, 0.1415]
28 clusters	0.1392	[0.1232, 0.1538]
29 clusters	0.1120	[0.0973, 0.1275]
30 clusters	0.1172	[0.1012, 0.1330]

Supplementary Table 5. Mean grid scores and bootstrap CIs in the trapezoid environment for each condition.

	Mean	bootstrap CIs
10 clusters	0.1998	[0.1754, 0.2234]
11 clusters	0.1918	[0.1749, 0.2096]
12 clusters	0.5556	[0.5300, 0.5807]
13 clusters	0.4931	[0.4671, 0.5162]
14 clusters	0.3924	[0.3687, 0.4185]
15 clusters	0.1110	[0.0869, 0.1365]
16 clusters	0.0550	[0.0301, 0.0760]
17 clusters	0.1818	[0.1551, 0.2073]
18 clusters	0.2865	[0.2599, 0.3132]
19 clusters	0.2670	[0.2427, 0.2900]
20 clusters	0.2619	[0.2386, 0.2869]
21 clusters	0.2066	[0.1851, 0.2283]
22 clusters	0.1856	[0.1628, 0.2068]
23 clusters	0.1549	[0.1309, 0.1779]
24 clusters	0.1398	[0.1183, 0.1615]
25 clusters	0.1587	[0.1360, 0.1832]
26 clusters	0.1549	[0.1335, 0.1775]
27 clusters	0.1479	[0.1231, 0.1680]
28 clusters	0.1470	[0.1241, 0.1693]
29 clusters	0.1666	[0.1448, 0.1874]
30 clusters	0.1377	[0.1170, 0.1594]

Supplementary Table 6. Mean grid difference scores and bootstrap CIs between the square and trapezoid environment for each condition.

	Mean	bootstrap CIs
10 clusters	0.0179	[-0.0128, 0.0444]
11 clusters	0.1503	[0.1263, 0.1765]
12 clusters	0.1270	[0.1063, 0.1492]
13 clusters	0.0965	[0.0786, 0.1151]
14 clusters	0.0784	[0.0540, 0.1013]
15 clusters	0.0767	[0.0491, 0.1089]
16 clusters	0.1451	[0.1141, 0.1784]
17 clusters	0.2387	[0.2069, 0.2691]
18 clusters	0.3434	[0.3106, 0.3761]
19 clusters	0.3020	[0.2723, 0.3361]
20 clusters	0.2424	[0.2131, 0.2700]
21 clusters	0.2161	[0.1875, 0.2423]
22 clusters	0.1515	[0.1237, 0.1768]
23 clusters	0.0999	[0.0746, 0.1278]
24 clusters	0.1108	[0.0848, 0.1388]
25 clusters	0.1122	[0.0834, 0.1392]
26 clusters	0.0789	[0.0508, 0.1055]
27 clusters	0.0635	[0.0359, 0.0940]
28 clusters	0.0714	[0.0421, 0.1013]
29 clusters	0.0197	[-0.0096, 0.0495]
30 clusters	0.0499	[0.0214, 0.0754]

Supplementary Table 7. Mean grid difference scores and bootstrap CIs between the wide and narrow portion of the trapezoid environment for each condition.

SUPPLEMENTARY REFERENCES

1. Krupic, J., Burgess, N. & O'Keefe, J. Neural representations of location composed of spatially periodic bands. *Science.* **337,** 853–857 (2012).

2. Perez-Escobar, J. A., Kornienko, O., Latuske, P., Kohler, L. & Allen, K. Visual landmarks sharpen grid cell metric and confer context specificity to neurons of the medial entorhinal cortex. *Elife* **5,** e16937 (2016).
